# Research on the construction of an evaluation index system of teachers’ online learning power based on online professional learning communities

**DOI:** 10.3389/fpsyg.2025.1586319

**Published:** 2025-09-03

**Authors:** Lei Xiang, Liumei Yu

**Affiliations:** School of Education, China West Normal University, Nanchong, China

**Keywords:** online professional learning communities, teacher, online learning power, professional development, evaluation index system

## Abstract

**Introduction:**

In line with the overarching demands of the current educational ecological reform, online learning should transition from “novelty” to “normalization.” Teachers in elementary and secondary schools are currently learning in an active style that stresses the development of learning power, gradually moving away from a passive form. The efficiency and effect of teachers’ online learning are closely correlated with their level of online learning power. However, teachers’ professional development is limited and there is a lack of direction at the practical level of online learning because there is no efficient evaluation index system for teachers’ online learning power. Therefore, this study first conducts an in-depth study on the ELLI project learning power theory and the online learning power structure framework based on the attributes of the online professional learning communities (OPLCs), such as autonomy, professionalism, and openness.

**Methods:**

On this basis, research methods including the Delphi method, the Analytic Hierarchy Process, and the questionnaire survey method are employed to study the evaluation dimensions of teachers’ online learning power. Establishing a scientific, logical, and operable index system for evaluating teachers’ online learning power is the aim. The OPLCs-based evaluation index system of teachers’ online learning power has been established following a series of studies that included the preliminary construction of the evaluation index system using the literature analysis method, the Delphi method for revision, the Analytic Hierarchy Process for determining the weights of each index item, and the questionnaire survey method for small-scale trial testing.

**Results:**

The evaluation index system consists of six first-level indicators and twenty-two second-level indicators. Furthermore, the evaluation index system’s dimension division and weight distribution are reasonable, well-scientific, and reliable.

**Discussion:**

This research offers vital support and decision-making basis for effective diagnosis and precise improvement of teachers’ online learning power, and puts forward new requirements and action guidelines for teachers’ professional development.

## 1 Introduction

The COVID-19 pandemic has compelled educational institutions all across the world to swiftly transition to remote teaching. According to a UNESCO survey on the COVID-19 pandemic’s effects on higher education, the rise in online education is the pandemic’s primary effect on teaching and learning. Online education has ushered in unprecedented development opportunities, accelerating its popularization. Even after the pandemic, distance teaching and blended teaching models will persist, and online learning will progressively emerge as a significant means of advancing teachers’ professional development (Yu et al., 2021). And online learning also will be an integral part of this new global educational landscape (Bragg et al., 2021).

Mr. Edgar Faure of UNESCO predicted: “The illiterate of the future will no longer be those who cannot read, but those who have not learned how to learn.” Additionally, according to Alvin Toffler, the illiterate of the 21st century will not be those who cannot read and write, but those who cannot learn, unlearn, and relearn (Toffler, 1970). Those who cannot learn are also those who have no learning power. Furthermore, the basis and premise of teachers’ effective online learning is online learning power, which is connected to the quality and efficiency of teachers’ online learning. As a result, the research on teachers’ online learning power is especially crucial.

Even if there have been many studies on the application evaluation of online learning power, teachers, who are special learners, have their unique professional characteristics, whereas the existing study objects are basically focused at general learners. As a result, the uniqueness of teachers’ power for professional learning is not adequately reflected in the research that are now available. This disparity prevents the current online learning power evaluation models from being widely used and promoted among teachers. Therefore, developing a suite of efficient online learning power evaluation index system designed especially for teachers is imperative.

Furthermore, a large number of studies have confirmed that OPLCs are essential platforms for teachers to engage in in-service training and achieve professional development. Teachers have achieved their all-round development in them. For instance, positive transformations in teachers’ motivation, engagement, and commitment to teaching within OPLCs (Alwafi et al., 2020; Zhang and Liu, 2019; Xing and Gao, 2018). Consequently, this study attempts to construct an OPLCs-based evaluation index system of teachers’ online learning power.

## 2 Literature review

### 2.1 Online professional learning communities (OPLCs)

There are varying degrees of interpretation in different national contexts (Stoll et al., 2006). For example, in China, professional learning communities (PLCs) for teachers are spontaneous organizations whose fundamental purpose is to enhance teachers’ professional capabilities and promote their professional development, and whose ultimate goal is common progress. However, it does exist a broad international consensus that the term “professional learning communities (PLCs)” suggests a group of people sharing and critically interrogating their practice in an ongoing, reflective, collaborative, inclusive, learning-oriented, growth-promoting way, and operating as a collective enterprise (Mitchell and Sackney, 2000; Toole and Louis, 2002; Stoll, 2004).

Hord (1997) listed several attributes of PLCs: supportive and shared leadership, collective creativity, shared values and vision, supportive conditions, and shared personal practice. Furthermore, Wang and Zhang (2023) stressed that effective professional development relies heavily on teachers’ consistent participation, active endorsement, and collective culture. González-Weil et al. (2025) proposed that in PLCs that are sustained over time, trust-building is a key element. Furthermore, through a longitudinal study, they describe how trust is built and maintained in a Chilean PLC of science teachers who meet regularly to share and reflect on their practice.

Because of PLCs, teachers become effective in their profession (Li, 2022). PLCs have emerged as a significant focus within the current research landscape concerning teacher professional development and learning (Yin and Qin, 2024). Forming PLCs has been recognized as a means to promote effective professional development (Wang and Zhang, 2023). PLCs are regarded as one of the most effective approaches for enhancing teachers’ professional development in the field of education (Zhou et al., 2022). They can facilitate the implementation of curriculum reform by fostering teacher professional development and enhancing teacher autonomy (Song and Wei, 2011). Professional development can be enhanced by establishing PLCs with digital tools. PLCs can be facilitated by moving conventional PLCs online or by reconstructing them as a hybrid or completely online form of meeting (Beach, 2012). Web-based teacher development programs and OPLCs have been highlighted as powerful professional development contexts due to the increased use of educational technology in online settings (Göktürk Sağlam and Dikilitaş, 2020). Teachers are increasingly using online communities for professional support, guidance, and inspiration, and they may be a source of ongoing professional development (Duncan-Howell, 2010). For example, a previous study has found that OPLCs can facilitate preservice teachers’ learning (Xie, 2022).

### 2.2 Online learning power framework

The concept of “learning power” was first proposed by Forrester in 1965 (Forrester, 1965), which became the central discourse of the learning organization in the field of management. Forrester believes that learning power is a comprehensive manifestation of an individual or organization’s motivation, perseverance and ability to learn. Among them, learning motivation stems from an individual’s curiosity and interest in unknown knowledge fields. Learning perseverance is a stable willpower quality formed by learners during the learning process. Learning ability is the internalized construction of knowledge acquired by learners during the learning process and the ability to solve problems, and it is a generalized experience formed under the influence of the environment and education. Following decades of development, it has emerged in the field of education and has become an important research topic in the field of pedagogy. In contrast, the learning power in the field of management mainly considers the improvement and transformation of the organizations through learning power, focuses on the study of organizational learning power, and influences individuals through organizational learning power. The research on learning power in the field of education mainly focuses on individual learning power. Due to different research perspectives, there are four positioning tendencies in the academic circle regarding the connotation of learning ability, namely the energy view, the character view, the quality view, and the ability view (Chen and Yang, 2010). According to most researchers, learning power is an energy, quality, accomplishment, or ability that is extremely important to the sustainable development of learners. It is objective and abstract, interacts with learning activities, and encompasses both dynamic processes and static consequences (Crick, 2007; Zheng and Xu, 2020; Zheng et al., 2025).

Optimizing online learning experiences and improving educational outcomes need the creation of online professional development programs for teacher educators (Zhao et al., 2024). Online learning power is a sub-concept of learning power that reflects the characteristics of online learning activities for distant learners in addition to having the attributes of general learning power (Ding et al., 2015; Zheng and Xu, 2020). Selim (2007) proposed that online learning power is a combination of learners’ learning motivation, information technology ability, collaborative interaction ability and learning attitude during the online learning process. Teja I and Spannaus (2008) held that online learning power is a dynamic ability system through which learners achieve learning results through various channels during the online learning process. McClendon et al. (2017) held that the online learning power of learners in online education is an individual quality integrating multiple factors, such as perseverance, etc. Li and Zhu (2017) held that the online learning power of online learners refers to the dynamic energy system that can effectively promote the interaction of learners’ learning motivation, cognitive ability, learning strategies and methods, and learning results in an online learning environment. It stimulates learners’ online learning motivation and potential, promotes learners to successfully complete online learning goals, and helps them achieve self-perfection and lifelong development.

Although the components of online learning power have not yet been unified, they mainly include the following aspects: the ability to adapt to the online learning context, the cognitive ability during knowledge learning, the ability to apply the knowledge after learning, and the ability to reflect, from which more elements are derived (Zheng et al., 2025). Teja and Spannaus (2008) held that the ability to use technology, the ability to collaborate, and the ability to manage time are important components of online learning ability. Hong and Jung (2011) constructed an online learning power evaluation model for successful distance learners using the structural equation model, including abilities such as learning vision, cognitive and metacognitive skills, interaction ability, learner identity, and management skills. Ding et al. (2015) constructed a theoretical model of distance learners’ online learning power, which is made up of four components: driven ability, willpower, cognition ability, and transformation ability. Li and Zhu (2017) separated online learning power into five dimensions: learning-driven ability, learning adaptation ability, learning response ability, learning management and adjustment ability, and learning reciprocity ability. Martin et al. (2020) held that students’ online learning power includes four abilities: online student attributes, time management, communication, and technology. Zheng and Xu (2020) proposed that online learning power mainly includes four dimensions: driven ability, cognition ability, willpower, and application ability. Hu and Liang (2022) divided online learning power into four parts: learning adaptation ability, learning cognition ability, learning application ability, and learning reflection ability. Wang et al. (2023) proposed that the online learning power theory model is a system composed of six dimensions: driven ability, adaptation ability, response ability, adjustment ability, transformation ability, and reciprocity ability. Zheng et al. (2025) classified online learning power into four perspectives: learning compliance ability, learning cognition ability, learning application ability, and learning reflection ability.

The goals of developing the assessment index system of teachers’ online learning capacity are summed up in this study. The following are the main objectives: (1) The evaluation index system of online learning power with teachers as the main body is constructed, which reflects the uniqueness of their professional learning power and make the evaluation index system more practical in the educational area. (2) OPLCs are used as a representative tool for instructors’ online learning in accordance with realistic circumstances. Consequently, an evaluation scale specifically designed to measure teachers’ online learning power based on OPLCs has been developed. This measure is used in practice as a guide to assess the online learning power. (3) The evaluation index system for online learning power in the field of vocational education is built by taking into account teachers’ general abilities as well as their professional competences. This initiative seeks to optimize teacher training mechanisms while advancing educational reform, and facilitating school transformation (Qiang and Zhang, 2018).

The following research issue and its sub-questions are addressed in this study: How can we construct an OPLCs-based evaluation index system of teachers’ online learning power?

Q1. Why is it necessary to construct it?

Q2. What is its precise content?

Q3. What is the application feedback like, and how scientific and reliable is it?

## 3 Materials and methods

**Figure 1:**
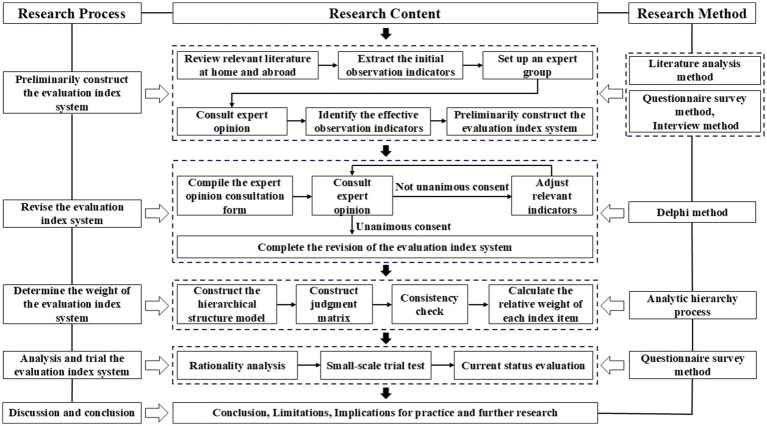
Research path diagram of the OPLCs-based evaluation index system of teachers’ online learning power.

The study path for developing the OPLCs-based evaluation index system of teachers’ online learning power is comprised of the subsequent steps, as illustrated in Figure 1.

### 3.1 Preliminary construction

**Figure 2:**
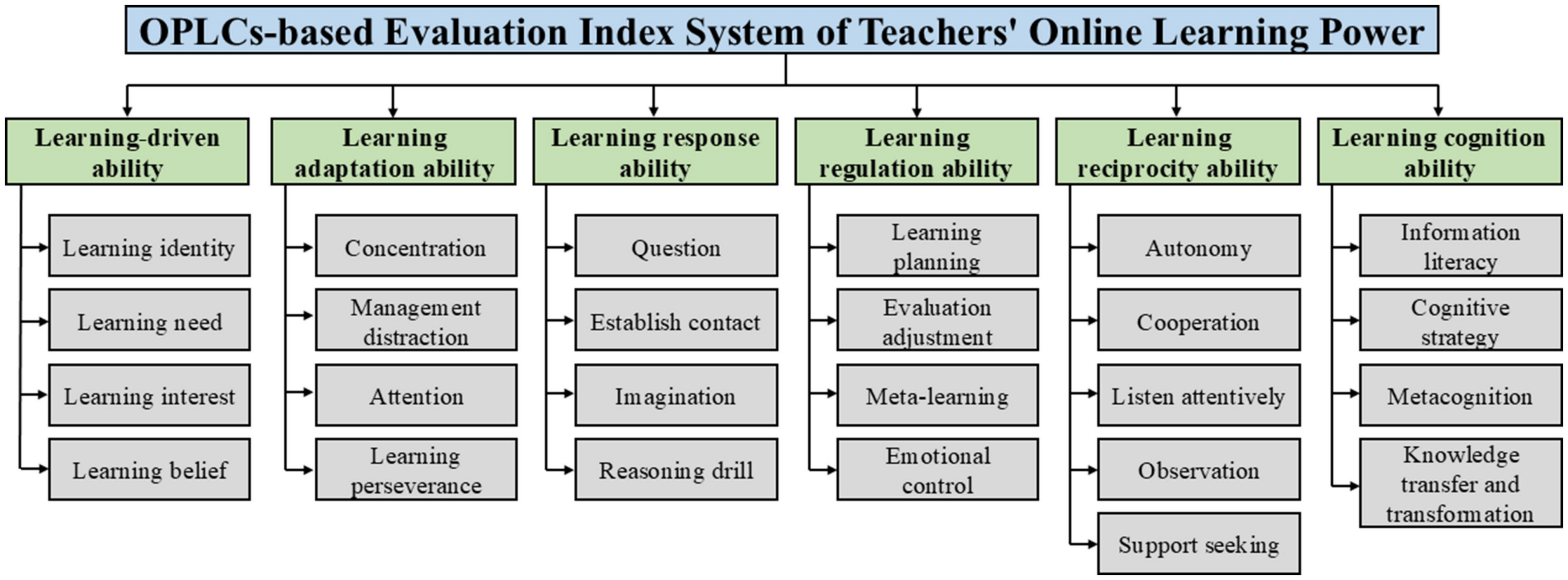
The preliminary construction diagram of the OPLCs-based evaluation index system of teachers’ online learning power.

This study derived the essential components and dimensions of the evaluation index system from the literature. An OPLCs-based evaluation index system of teachers’ online learning power was initially constructed in conjunction with expert consultation. As shown in Figure 2, it included six first-level indicators and twenty-five second-level indicators. According to this study, the driving force of carrying out online learning (learning-driven ability), the ability to adhere to online learning (learning adaptation ability), the ability to employ learning strategies (learning response ability), the ability to regulate the learning process (learning regulation ability), the ability to seek collaboration and mutual gain (learning reciprocity ability), and the ability to master knowledge and skills (learning cognition ability) are all components of teachers’ online learning power.

### 3.2 Revision

#### 3.2.1 First round of expert consultation

Key data including each indicator’s mean value of importance, standard deviation, full score frequency, and coefficient of variation were computed using the EXCEL16.0 software. The evaluation index system was revised using these statistics as the quantitative basis and expert feedback as the qualitative basis ensured that the revision process was reasonable, scientific, and evidence-based. The degree of concentration of experts’ opinions was reflected in the mean value of importance and the full score frequency. The mean value of importance refers to the arithmetic mean of the importance score of an indicator by the expert. The greater the mean value, the greater the importance of the indicator in the index system. The full score frequency refers to the ratio of the number of experts giving a full score to the total number of experts participating in the score of an indicator. The greater the full score frequency, the greater the importance of the indicator in the index system. The coefficient of variation is defined as the ratio of the standard deviation of a specific indicator to its mean value. The coefficient of variation indicates the degree of fluctuation in experts’ evaluations regarding the importance of a certain indicator, which can reflect the consistency of the experts’ judgment opinions on an item, or whether there are disagreements and the degree of disagreement. The smaller the coefficient of variation, the higher the degree of consensus among experts.

Two rounds of expert consultations were conducted in this study. A total of 11 experts were selected, including 7 experts in the field of educational technology and 4 experts in the field of education. In each round, 11 expert consultation questionnaires were issued, and 11 valid questionnaires were retrieved, with a recovery rate of 100%. The mean value of importance larger than or equal to 4 and the coefficient of variation less than or equal to 0.2 are the criteria for screening indicators in this study. Table 1 displays each indicator’ score from the first round of expert consultation. Among the six first-level indicators, the coefficient of variation does not exceed 0.2, and the mean value of the importance of the other five first-level indicators is not less than 4, with the exception of ‘learning response ability’, which has the mean value of the importance of less than 4. We conducted further expert discussions based on the data analysis, and we contrasted our findings with those of previous studies. In the end, we made the decision to keep the “learning response ability” indicator while modifying the second-level indicator that goes with it; as a result, all first-level indicators were kept. Seven of the twenty-five second-level indicators—'concentration’, ‘learning perseverance’, ‘question’, ‘establish contact’, ‘reasoning drill’, ‘evaluation adjustment’, and ‘meta-learning’—have the mean value of importance that are less than 4. Additionally, both ‘attention’ and ‘reasoning drill’ demonstrate the coefficient of variation exceeding 0.2. The remaining seventeen indicators maintain the mean value of importance no less than 4 along with the coefficient of variation not surpassing 0.2. As a result, these eight indicators must be revised based on expert feedback gathered from the open-ended questions in the questionnaire. The responses to these open-ended questions make it evident that most professionals think the dimensions and structure of the evaluation index system are logical and unambiguous. Four main points are the main emphasis of the expert opinions: (1) The indicators of “imagination” and “reasoning drill” within the “learning response ability” category share semantic similarities. Can these two indications be combined? (2) Should the ‘concentration’ indicator be removed, considering that online learning materials can be paused and reviewed? (3) There appears to be a redundancy between the ‘attention’ and ‘concentration’ indicators within the ‘learning adaptation ability’ category. Can these also be merged? (4) Are there too many evaluation dimensions, and could some similar dimensions be consolidated?

**Table 1:** Results of the first round of expert consultation.

First-level indicators	Mean value of importance	Standard deviation	Full score frequency	Coefficient of variation	Second-level indicators	Mean value of importance	Standard deviation	Full score frequency	Coefficient of variation
Learning-driven ability	4.30	0.31	0	0.07	Learning identity	4.18	0.40	0.18	0.10
					Learning need	4.18	0.75	0.36	0.18
					Learning interest	4.45	0.52	0.45	0.12
					Learning belief	4.36	0.50	0.36	0.12
Learning adaptation ability	4.00	0.38	0	0.10	Concentration	3.91	0.54	0.09	0.14
					Management distraction	4.18	0.60	0.27	0.14
					Attention	4.18	0.87	0.45	0.21
					Learning perseverance	3.64	0.67	0	0.19
Learning response ability	3.84	0.52	0	0.13	Question	3.55	0.69	0	0.19
					Establish contact	3.91	0.70	0.18	0.18
					Imagination	4.18	0.60	0.27	0.14
					Reasoning drill	3.82	0.87	0.18	0.23
Learning regulation ability	4.05	0.53	0	0.13	Learning planning	4.00	0.77	0.27	0.19
					Evaluation adjustment	3.91	0.54	0.09	0.14
					Meta-learning	3.91	0.70	0.18	0.18
					Emotional control	4.36	0.67	0.45	0.15
Learning reciprocity ability	4.11	0.36	0	0.09	Autonomy	4.00	0.63	0.18	0.16
					Cooperation	4.18	0.60	0.27	0.14
					Listen attentively	4.18	0.60	0.27	0.14
					Observation	4.09	0.54	0.18	0.13
					Support seeking	4.09	0.54	0.18	0.13
Learning cognition ability	4.34	0.45	0.18	0.10	Information literacy	4.45	0.52	0.45	0.12
					Cognitive strategy	4.09	0.70	0.27	0.17
					Metacognition	4.36	0.81	0.55	0.19
					Knowledge transfer and transformation	4.45	0.52	0.45	0.12

In response to Opinion (1), this study integrates ‘imagination’ and ‘reasoning drill’ under the ‘learning response ability’ indicator, rephrasing them as ‘reasoning argumentation’. This involves semantically consolidating C31 (Able to clarify the knowledge structure and think about the composition of the curriculum system), C32 (Able to use imagination and multiple senses to learn and try different learning methods), C41 (Able to apply what you have learned to practice and think about rehearsals or drills in your mind), and C42 (Able to predict what will happen and practice action steps in your mind). The revised statements are: ‘Be able to think about different ways of learning and rehearse or rehearse specific application steps in your mind beforehand’, ‘When learning online, I can predict what will happen’, and ‘When encountering problems, I can reason out a better strategy to solve the problem’.

In response to Opinion (2), a growing number of academics have shown a strong link between learning performance and concentration levels, with this association being especially noticeable in online learning contexts. Consequently, this study retains the ‘concentration’ indicator under the ‘learning adaptation ability’ indicator.

In response to Opinion (3), experts have discussed that there is a semantic duplication between B31 (Ability to selectively accept and absorb course content, focus on learning focus) and the dimension of ‘learning response ability’. There exists a semantic duplication between B32 (Ability to patiently find solutions to problems and pay attention to details) and the dimension of ‘learning perseverance’. Consequently, the ‘attention’ indicator has been removed.

In response to Opinion (4), regarding C12 (Be willing to ask questions or things unknown in the online professional learning communities) under the ‘question’ indicator, expert discussions revealed that it has a semantic ambiguity with both the ‘cooperation’ and ‘support seeking’ indicators, therefore, C12 has been deleted. Regarding B43 (Be able to consciously overcome inertia and bad habits in online learning) within the context of the ‘learning perseverance’ indicator, the expert group determined that it more appropriately belongs under ‘learning attitude and habits’. And there is also semantic ambiguity between this indicator and A42 (Ability to work hard for online learning and seriously complete each learning task), therefore, B43 has been deleted. For the indicator concerning ‘evaluation adjustment’, there is a semantic duplication between D21 (Be able to compare online learning results with expected goals, find problems, and adjust learning plans in a timely manner) and D22 (Be able to flexibly adjust learning plans or change directions according to actual conditions). Therefore, these two indicators have been combined into one: ‘According to the actual situation of online learning, I can find problems, adjust the learning plan, or change the direction in time’.

The mean value of importance of the two second-level indicators, ‘establish contact’ and ‘meta-learning’ is getting close to 4, while the coefficient of variation is still less than 0.2. This indicates that the expert’s evaluation of their importance tends to be ‘important’ with a high degree of consensus. Following further discussions with experts along with comparative analyses aligned with existing research findings, it was ultimately decided that these two indicators would be retained. As such, after completing the first round of revisions, the evaluation index system currently comprises six first-level indicators alongside twenty-three second-level indicators.

#### 3.2.2 Second round of expert consultation

The revised evaluation index system from the first round served as the basis for the second round’s expert consultation questionnaire. According to the findings of the second round of expert consultations, the coefficient of variation has greatly decreased (all below 0.2), and the mean value of importance of each indicator has significantly increased (all above 4.0). The outcomes of the second round of expert consultations for each indicator are shown in Table 2.

**Table 2:** Results of the second round of expert consultation.

First-level indicators	Mean value of importance	Standard deviation	Full score frequency	Coefficient of variation	Second-level indicators	Mean value of importance	Standard deviation	Full score frequency	Coefficient of variation
Learning-driven ability	4.14	0.26	0	0.06	Learning identity	4.09	0.70	0.27	0.17
					Learning need	4.09	0.30	0.09	0.07
					Learning interest	4.18	0.75	0.36	0.18
					Learning belief	4.27	0.65	0.36	0.15
Learning adaptation ability	4.24	0.40	0.09	0.09	Concentration	4.09	0.54	0.18	0.13
					Management distraction	4.27	0.47	0.27	0.11
					Learning perseverance	4.09	0.54	0.18	0.13
Learning response ability	4.21	0.52	0.18	0.12	Question	4.27	0.79	0.45	0.18
					Establish contact	4.27	0.65	0.36	0.15
					Reasoning argumentation	4.27	0.65	0.36	0.15
Learning regulation ability	4.20	0.27	0	0.06	Learning planning	4.09	0.54	0.18	0.13
					Evaluation adjustment	4.55	0.52	0.55	0.11
					Meta-learning	4.18	0.60	0.27	0.14
					Emotional control	4.09	0.83	0.36	0.20
Learning reciprocity ability	4.29	0.44	0.18	0.10	Autonomy	4.00	0.63	0.18	0.16
					Cooperation	4.36	0.67	0.45	0.15
					Listen attentively	4.18	0.40	0.18	0.10
					Observation	4.45	0.52	0.45	0.12
					Support seeking	4.18	0.60	0.27	0.14
Learning cognition ability	4.27	0.49	0.18	0.12	Information literacy	4.36	0.50	0.36	0.12
					Cognitive strategy	4.27	0.65	0.36	0.15
					Metacognition	4.36	0.81	0.55	0.19
					Knowledge transfer and transformation	4.09	0.70	0.27	0.17

**Figure 3:**
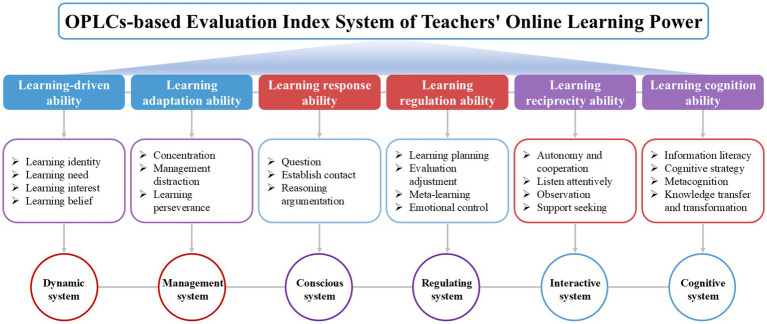
The OPLCs-based evaluation index system of teachers’ online learning power.

In comparison to the overall framework, experts voiced concern about the excessive number of second-level indicators pertaining to “learning reciprocity ability” and recommended that some indications be combined. It was finally determined to combine the “autonomy” and “cooperation” indicators into a single “autonomy and cooperation” indicator after further expert discussions and integration of previous study findings. It is clear that the outcomes of this second round of expert consultation have also come closer to agreement based on both index data and open feedback. As a result, the third round of expert consultation is no longer held. As shown in Figure 3, the revised evaluation index system after the second round of expert consultation thus consists of six first-level indicators and twenty-two second-level indicators.

### 3.3 Determine the weight

Each indicator must be given a weight in order to improve the practicality of this evaluation index system. To determine these weights, we choose to use the analytic hierarchy process (AHP) and AHP software, yaahp, in accordance with the real situation. An ‘Evaluation Index System Weight Scoring Table’ has been compiled for collecting expert assessments regarding weight allocations across all levels of indicators. The following describes the precise procedures involved in the calculation.

#### 3.3.1 Construct the hierarchical structure model

**Figure 4:**
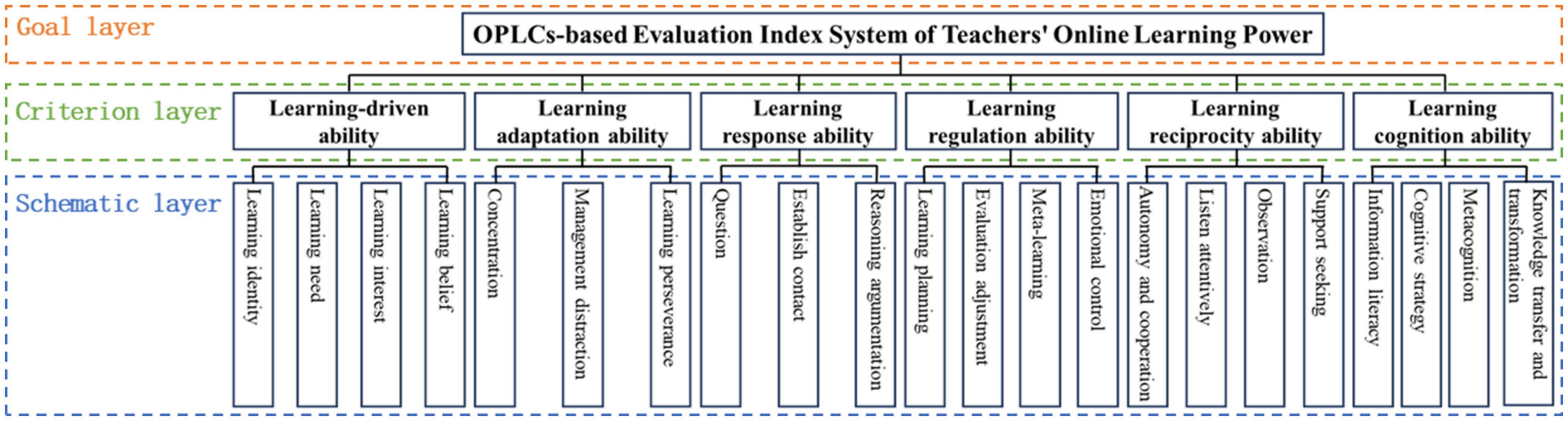
Hierarchical structure model of the OPLCs-based evaluation index system of teachers’ online learning power.

The first step is to use yaahp software to construct a hierarchical structure model of the OPLCs-based evaluation index system of teachers’ online learning power, as shown in Figure 4.

#### 3.3.2 Construct judgment matrix

Secondly, the judgment matrix corresponding to each indicator has been constructed. The relative importance scores of eleven experts have been processed by geometric mean. Then these processed scores were subsequently incorporated into the judgment matrix. Take the judgment matrix of the first-level indicators of online learning power as an example, as shown in Table 3.

**Table 3:** Judgment matrix of the first-level indicators of online learning power.

Indicators	Learning-driven ability	Learning adaptation ability	Learning response ability	Learning regulation ability	Learning reciprocity ability	Learning cognition ability
Learning-driven ability	1	5.1150	4.5867	3.3629	5.1568	1
Learning adaptation ability		1	0.5004	0.4624	0.9050	0.1855
Learning response ability			1	1.0537	2.0906	0.2291
Learning regulation ability				1	2.0882	0.2279
Learning reciprocity ability					1	0.2078
Learning cognition ability						1

#### 3.3.3 Consistency check

Thirdly, the consistency of the judgment matrix is examined. The maximum eigenvalue (𝜆𝑚𝑎𝑥) is computed using Equation (1). The appropriate average random consistency index (RI) values are determined by the matrix’s order: RI = [0, 0, 0.52, 0.89, 1.12, 1.26]. The consistency index (CI) values are computed using Equation (2). The random consistency ratio (CR) values are then computed using Equation (3). The consistency test results for the judgment matrix are shown in Table 4. The results demonstrate that the CR values of each judgment matrix are notably less than 0.1, meeting the matrices’ overall consistency requirements and proving that their consistency is adequate.

(1) $$\text{λmax}=\sum \frac{{\left(\mathrm{AW}\right)}_{i}}{{\mathrm{nw}}_{i}}$$

(2) $$\mathrm{CI}=\frac{\text{λmax}-n}{n-1}$$

(3) $$\mathrm{CR}=\frac{\mathrm{CI}}{\mathrm{RI}}$$

**Table 4:** Results of consistency test of judgment matrix.

Judgment matrix	𝜆𝑚𝑎𝑥	CI	RI	CR	Consistency test results
Online learning power	6.073	0.015	1.260	0.012	PASS
Learning-driven ability	4.006	0.002	0.890	0.002	PASS
Learning adaptation ability	3.000	0.000	0.520	0.000	PASS
Learning response ability	3.002	0.001	0.520	0.002	PASS
Learning regulation ability	4.018	0.006	0.890	0.007	PASS
Learning reciprocity ability	4.013	0.004	0.890	0.005	PASS
Learning cognition ability	4.005	0.002	0.890	0.002	PASS

#### 3.3.4 Calculate the relative weight

Finally, after verifying that all judgment matrices meet consistency criteria, weights for six first-level indicators and twenty-two second-level indicators are calculated using an intelligent algorithm within the yaahp software to complete the construction of the evaluation index system, as shown in Table 5.

**Table 5:** The OPLCs-based evaluation index system of teachers’ online learning power.

First-level indicators	Relative weights	Second-level indicators	Relative weights	Indicator connotations
Learning-driven ability	0.3367	Learning identity	0.2092	Ability to hold behavioral identity awareness of online learning behavior
				Ability to hold a sense of value recognition for the change of learning behavior
				Ability to hold a sense of result recognition for online learning results
		Learning need	0.2962	Ability to know what you really need to learn
				Ability to correctly perceive the gap between the existing level and the expected level
		Learning interest	0.2562	Ability to actively and intensively study online courses of interest
				Ability to study in-depth and research topics of interest
		Learning belief	0.2384	Ability to firmly believe that online learning helps to achieve professional development and achieve learning goals
				Ability to work hard for online learning and seriously complete each learning task
Learning adaptation ability	0.0556	Concentration	0.3987	Ability to focus and block out external distractions
				It feels good to be able to feel truly engaged in online learning
		Management distraction	0.1651	Ability to recognize and manage distractions and know what needs to be adjusted
				Ability to redirect distracted attention to online learning
				Be able to know what conditions can help and promote their deep learning
				Be able to know when you can stop to adjust to take a break
		Learning perseverance	0.4362	Knowing that online learning means working tirelessly and challenging, but still hopeful
				Able to persist and find solutions to problems, and will not give up easily
Learning response ability	0.0978	Question	0.3426	Can be good at asking questions, to guide their online learning
		Establish contact	0.4187	The ability to establish cognitive diagrams for related knowledge or things, forming cognitive network diagrams
				Ability to relate content points to existing knowledge, experience, and professional practice
				Able to construct the picture of knowledge and application, and connect the theory, principle, and application situation
		Reasoning argumentation	0.2388	Be able to think about different ways of learning and rehearse or rehearse specific application steps in your mind beforehand
				When learning online, I can predict what will happen
				When encountering problems, I can reason out a better strategy to solve the problem
Learning regulation ability	0.1024	Learning planning	0.2969	Be able to develop a clear learning plan, divided into specific learning objectives
				Ability to manage time and schedule steps according to learning objectives and content
				For different learning tasks, I can know when to use which way of thinking or learning strategies
		Evaluation adjustment	0.1277	According to the actual situation of online learning, I can find problems, adjust the learning plan, or change the direction in time
				Be willing to adjust my learning schedule if I have better ideas or practices
		Meta-learning	0.3642	Ability to communicate with peers how to learn online and think about effective learning methods
				As an online learner, I can know your advantages and disadvantages.
				Be able to communicate with peers about online learning practices and share harvest feelings
				Be able to perform well as a team member
		Emotional control	0.2112	Be able to maintain a positive mood and be patient with online learning
				It can relieve negative emotions and resist rejection from within the individual
				It can maintain positive emotions, make it develop healthily and happily for life, and improve the psychological happiness index
Learning reciprocity ability	0.0588	Autonomy and cooperation	0.5360	In online learning, I can know when and what kind of situation is suitable for autonomous learning
				Be able to hold my own opinions and stand my ground in cooperative learning
				In online learning, I can know when and what kind of situation is suitable for cooperative learning
				In cooperative learning, I can know how to manage myself, respect the views of others, and am willing to share my views with others
		Listen attentively	0.1719	In cooperative learning, I can listen carefully to the views of others
				When others express their opinions, I am able to think about the position of the other person’s opinion
				Be able to understand the intentions of others, easy to stand in the position of others to see the problem, with empathy
		Observation	0.1059	Be able to pay attention to others and learn from their good learning methods or practices
				Being able to learn mindfully when others show how to learn or apply what they have learned
		Support seeking	0.1863	Be able to actively seek support and help from others when encountering difficulties
				Be able to actively provide support and help when others are in trouble
Learning cognition ability	0.3487	Information literacy	0.2827	Have the corresponding information technology knowledge and information technology skills, can use online professional learning communities for online learning, cooperation, and communication
				Have the corresponding information consciousness and social ethics consciousness, and can comply with the rules and regulations of the online professional learning communities
		Cognitive strategy	0.2615	Be able to process and organize information effectively and store it systematically
				Ability to acquire relevant knowledge and skills through online learning
		Metacognition	0.3709	Ability to plan, monitor, reflect, and evaluate my own online learning process
				Be able to adjust and improve my cognitive processes
		Knowledge transfer and transformation	0.0849	Be able to consciously transfer the results of online learning into teaching practice
				Be able to consciously translate the results of online learning into practical results of teaching

## 4 Results

### 4.1 Rationality analysis

#### 4.1.1 Dimension division

This study takes ‘online learning power’ as the starting point and integrates it with teachers’ professional characteristics to determine evaluation dimensions related to teachers’ online learning power. These dimensions include learning-driven ability, learning adaptation ability, learning response ability, learning regulation ability, learning reciprocity ability, and learning cognition ability. Learning cognition ability is a “cornerstone” for successful online learning and is both a requirement and a basis for teachers to implement online learning. Learning-driven ability serves as an ‘engine’ that drives online learning ahead and cultivates intrinsic motivation among teachers. Learning adaption ability acts as a protective “shield” in the online learning, enabling teachers to navigate intelligent learning settings while removing outside distractions. Learning response ability serves as the ‘knowledge capsule’ of online learning, enabling teachers to use a wide range of learning techniques. As the “regulator” of online learning, learning regulation ability enables teachers to adapt online instruction flexibly to current conditions and practical demands. Learning reciprocity ability serves as the “bridge” for online learning by enabling connections between students, creating a strong bond marked by cooperation and support, and creating a learning community. The comprehensive ability framework of teachers’ online learning power is made up of these six dimensions, which are the essential abilities of their online learning. They are interrelated, work together on teachers’ online learning process, and affect the quality of teachers’ online learning.

#### 4.1.2 Weight distribution

The weight of an indicator reflects its importance within its respective levels or across the overall index system (Wu, 2009). In this study, we determine the weights given to each indication in the framework for evaluating teachers’ online learning power using the analytic hierarchy process. The first-level dimensions are listed below in descending order of importance: learning cognition ability, learning-driven ability, learning regulation ability, learning response ability, learning reciprocity ability, learning adaptation ability. This weight distribution seems appropriate in light of the current integrated development of digital technology and schooling. Notably, learning cognition ability is both fundamental to and at the heart of teachers’ online learning power, which is seen to be the most significant of them. Second in importance is learning-driven ability, which stands for intrinsic motivation, a crucial component affecting a person’s involvement in online learning. Learning response ability and regulation ability are examples of execution and strategy-level skills, which are less significant but are crucial for raising the effectiveness and standard of online education. Learning reciprocity ability and learning adaptation ability pertain to learners’ social interactions and environmental adaptability, making them the least important among these abilities.

### 4.2 Small-scale trial test

#### 4.2.1 Questionnaire preparation and investigation

It is crucial to further evaluate the evaluation index system’s alignment with the present state of teachers’ online learning power development once it has been established. To this end, a small-scale trial was carried out to test the evaluation index system’s reliability and scientificity. The questionnaire, named ‘Questionnaire on Teachers’ Online Learning Power Based on Online Professional Learning Communities’, was created based on the index’s connotations. The questionnaire is divided into two sections: the first collects basic information on teachers using eight items, and the second piece uses fifty-five items to evaluate teachers’ online learning power. According to the method of stratified sampling, front-line teachers (elementary and secondary school teachers) of different genders, ages, teaching years, professional titles, educational qualifications, teaching subjects and working units were selected as the research subjects, and a total of 156 valid questionnaires were collected. Based on odd and even numbers, the data from these legitimate questionnaires were separated into two almost identical datasets. Item analysis and exploratory factor analysis were conducted using one dataset (N1 = 78), whereas the reliability test was conducted using the other dataset (N2 = 78).

#### 4.2.2 Item analysis

Finding out if there is differentiation among the different items in the questionnaire is the goal of item analysis. We first determined each respondent’s overall score for all items in the questionnaire’s online learning power assessment section, then ranked the results from highest to lowest. Specifically speaking, those in the top 27% constituted what we termed as ‘the high group’, while those in the behind 73% formed ‘the low group’. Specific crucial points related to these scores were calculated using SPSS version 26.0. In this study, ‘the low group’ is defined as those with scores below 202, and ‘the high group’ is defined as those with scores above 240. Table 6 displays the scores’ crucial points. Specific crucial points related to these scores were calculated using SPSS version 26.0. In this study, ‘the low group’ is defined as those with scores below 202, and ‘the high group’ is defined as those with scores above 240. Table 6 displays the scores’ crucial points. To determine whether there were significant differences between the low and high groups on each item, the independent samples t-test was then employed. The findings show that each item’s *p*-value (two-tailed) is less than 0.001, indicating that a differentiation among the items. Through item analysis, a total of 55 items were retained.

**Table 6:** Scores critical points.

Percentile	Score
27% (the high group)	240
73% (the low group)	202

#### 4.2.3 Exploratory factor analysis (EFA)

SPSS version 26.0 was used for EFA. To determine whether EFA is appropriate, the questionnaire items were first subjected to the KMO test and the Bartlett Spherical Test. The findings revealed a KMO value of 0.794, and Bartlett’s Sphericity Test (*X*^2^ = 5123.349, df = 1,485, *p* = 0.000) indicated significant correlations among the questionnaire items, confirming its appropriateness for EFA. Based on these results, EFA was executed with principal component analysis employed to extract common factors. The eigenvalue larger than one was used to calculate the number of factors. For the orthogonal rotation axis, the maximum variance approach was applied; items were screened if their loading difference between two factors was less than 0.2 and their factor loadings were less than 0.4. According to the above criteria and methods, the items should be screened. Based on retaining at least two items per dimension as far as possible, experts reviewed these screening results to decide whether to delete the abnormal items. In the end, fifty things were kept, but five (QD13, SY23, CY32, TJ13, and TJ32) were eliminated. Ultimately, this study yielded six common components with a 73.052 percent cumulative variance interpretation rate.

#### 4.2.4 Reliability test

SPSS version 26.0 was used to examine index reliability since reliability refers to the consistency and dependability of the evaluation scale. It is generally accepted that a Cronbach’s *α* coefficient greater than 0.8 indicates high reliability. Table 7 displays the reliability test findings for online learning power overall and its first-level indicators. The findings showed that the Cronbach’s α coefficients for the entire scale of online learning power and the six first-level dimensions all surpassed 0.8. This result indicates that the scale has good internal consistency and a high degree of overall reliability. Together with testing the index system, the author also collected teacher input on the system. The evaluation index system’s scientific validity and reliability are confirmed by the majority of respondents who said they thought the system’s design was logical and its weight distribution was sound. This makes it a strong instrument for evaluating teachers’ online learning power.

**Table 7:** Reliability test results of online learning power as a whole and its first-level indicators.

Dimension	Number of items	Cronbach’s α coefficient
Learning-driven ability	9	0.924
Learning adaptation ability	8	0.920
Learning response ability	7	0.911
Learning regulation ability	12	0.947
Learning reciprocity ability	11	0.951
Learning cognition ability	8	0.940
Online learning Power	55	0.986

### 4.3 Current status evaluation

According to the established evaluation index system, specific content items under each second-level indicator are allocated proportionately and each second-level indicator is given a total score of 100 points. Teachers’ self-assessment of their online learning power was categorized into five levels using a percentile evaluation scale. From the lowest to the highest, ‘very inconsistent’, ‘not quite consistent’, ‘general’, ‘basically consistent’, and ‘very consistent’. These categories were, respectively, allocated scores of 20 points, 40 points, 60 points, 80 points, and 100 points. Teachers evaluate their current level of online learning power by determining how well they have met the standard. To determine the value of the first-level indicator to which the second-level indicator belongs, multiply each second-level indicator’s score by its weight and then sum all the values of the same first-level indicator. The current level of teachers’ online learning power is then determined by multiplying the value of each first-level indicator by its weight (Lin et al., 2020).

It is clear from calculating the scale’s results that the 156 teachers who took part in the survey had an average level of online learning power of 79.96 points, which suggests a relatively good level of online learning power. According to their self-evaluations, teachers exhibit a high degree of recognition regarding their own ‘learning-driven ability’ and ‘learning reciprocity ability’. This is followed by ‘learning adaptation ability’, ‘learning regulation ability’, and ‘learning cognition ability’. However, teachers perceive their ‘learning response ability’ as relatively insufficient. These results suggest that although the majority of teachers have the necessary core knowledge and skills and are eager to participate in online learning, they lack effective strategies to deal with online learning. Therefore, bolstering this specific sector should be a priority in the future development of online learning power.

Furthermore, lower scores accompanied by high standard deviations in ‘meta-learning’ and ‘emotional control’ indicate significant individual differences among teachers in these dimensions; there exists polarization within these abilities which do not meet ideal standards overall. The low scores and standard deviations found in domains like “question,” “establish contact,” and “reasoning argumentation” indicate that teachers generally perform poorly across these three ability dimensions, indicating that the problem is widespread rather than unique to any one person. This further corroborates the findings that teachers’ levels of ‘learning response ability’ are notably deficient. According to interviews, this predicament results from a pronounced conflict between teachers’ work and opportunities for professional development through learning. Teachers usually have little experience with online learning and lack comprehensive training in the area. Similar circumstances exist for other individual metrics as well; these will not be restated here.

## 5 Discussion and conclusion

### 5.1 Discussion

Even though there have been many studies on the application evaluation of online learning power before, the research subjects have basically focused on general learners, and the research subjects are relatively general and broad. Although teachers also fall within the scope of learners, as special learners, teachers should have their unique professional characteristics. Therefore, in the existing research on online learning power, the uniqueness of teachers’ professional learning power has not been fully reflected. However, in this study, teachers were taken as the research objects and a set of online learning ability evaluation index systems was specially developed for them. It is more targeted and operational compared with the existing studies. In comparison to relevant studies, the evaluation index system developed in this paper exhibits both similarities and distinctions. For instance, online learning power is categorized into six first-level dimensions: learning-driven ability, learning adaptation ability, learning response ability, learning regulation ability, learning reciprocity ability, and learning cognition ability. This categorization aligns closely with existing assessments of online learning power. However, the second-level dimensions—such as ‘emotional control’, ‘metacognition’, and ‘knowledge transfer and transformation’, among other indicators—emphasize the professional specificity of the subject. It reflects contemporary characteristics pertinent to teacher training in the new era while placing greater emphasis on aspects such as personal happiness, sense of acquisition, and sustainable development ability.

### 5.2 Conclusion

This study takes teachers as the main body, combined with the professional particularity of teachers, etc., and constructs the scientific and reliable OPLCs-based evaluation index system of teachers’ online learning power that comprises six first-level indicators and twenty-two second-level indicators. It is an essential tool for assessing the level and stage of developmental characteristics of teachers’ online learning power. It facilitates a clear understanding of the current status, potential, and developmental trends in teachers’ online learning power, thereby enhancing the quality of their online learning and promoting teaching reform. It will enhance the scientificity and systematicness of theoretical research on online learning power, and offer vital support and decision-making basis for effective diagnosis and precise improvement of teachers’ online learning power.

### 5.3 Limitations

Nonetheless, this study does have some deficiencies. Despite undergoing a rigorous literature review process and expert consultations during the construction of the evaluation index system for online learning power, there is not yet much consensus in the research field of teachers’ online learning power. Thus, the reference value of this research is limited. Consequently, the evaluation index system constructed in this study has some issues, such as uneven division of index items, uneven granularity of expression, and difficulty in understanding.

### 5.4 Implications for practice and further research

The optimization of expert group composition during the screening and integration of observation indicators should be the main focus of subsequent research. This optimization should involve a larger number and a broader range of experts to enhance the objectivity and scientific rigor in constructing the evaluation index system. Furthermore, it is noted that the sample size during the trial phase was insufficient, which may lead to inadequate explanatory power. In future endeavors, we will try to carry out continuous evaluation practices on a larger scale and in more regions to constantly improve the evaluation index system.

## Data Availability

The original contributions presented in the study are included in the article/Supplementary material, further inquiries can be directed to the corresponding author.
