# Supplementary material for: Research on the construction of an evaluation index system of teachers’ online learning power based on online professional learning communities
Source: Front Psychol. 2025 Sep 3;16:1586319. doi: 10.3389/fpsyg.2025.1586319 (PMC12440714; doi:10.3389/fpsyg.2025.1586319)
Supplement: Supplementary file 1 [file Supplementary_file_1.docx]

Supplementary Material

# Supplementary Tables

## Supplementary Figures

**Supplementary Figure 1.** Learning-driven ability judgment matrix

| Indicators | Learning identity | Learning need | Learning interest | Learning belief |
| --- | --- | --- | --- | --- |
| Learning identity | 1 | 0.6862 | 0.7529 | 0.9767 |
| Learning need |  | 1 | 1.1576 | 1.2126 |
| Learning interest |  |  | 1 | 0.9930 |
| Learning belief |  |  |  | 1 |

**Supplementary Figure 2.** Learning adaptation ability judgment matrix

| Indicators | Concentration | Management distraction | Learning perseverance |
| --- | --- | --- | --- |
| Concentration | 1 | 2.4397 | 0.9050 |
| Management distraction |  | 1 | 0.3822 |
| Learning perseverance |  |  | 1 |

**Supplementary Figure 3.** Learning response ability judgment matrix

| Indicators | Question | Establish contact | Reasoning argumentation |
| --- | --- | --- | --- |
| Question | 1 | 0.8557 | 1.3719 |
| Establish contact |  | 1 | 1.8349 |
| Reasoning argumentation |  |  | 1 |

**Supplementary Figure 4.** Learning regulation ability judgment matrix

| Indicators | Learning planning | Evaluation adjustment | Meta-learning | Emotional control |
| --- | --- | --- | --- | --- |
| Learning planning | 1 | 2.4586 | 0.8777 | 1.2502 |
| Evaluation adjustment |  | 1 | 0.3080 | 0.7182 |
| Meta-learning |  |  | 1 | 1.6489 |
| Emotional control |  |  |  | 1 |

**Supplementary Figure 5.** Learning reciprocity ability judgment matrix

| Indicators | Autonomy and cooperation | Listen attentively | Observation | Support seeking |
| --- | --- | --- | --- | --- |
| Autonomy and cooperation | 1 | 3.3471 | 4.3482 | 3.1449 |
| Listen attentively |  | 1 | 1.7796 | 0.9050 |
| Observation |  |  | 1 | 0.5280 |
| Support seeking |  |  |  | 1 |

**Supplementary Figure 6.** Learning cognition ability judgment matrix

| Indicators | Information literacy | Cognitive strategy | Metacognition | Knowledge transfer and transformation |
| --- | --- | --- | --- | --- |
| Information literacy | 1 | 1.1050 | 0.8126 | 3.0525 |
| Cognitive strategy |  | 1 | 0.6754 | 3.2944 |
| Metacognition |  |  | 1 | 4.4799 |
| Knowledge transfer and transformation |  |  |  | 1 |
